# A new X-ray images enhancement method using a class of fractional differential equation

**DOI:** 10.1016/j.mex.2023.102264

**Published:** 2023-06-22

**Authors:** Rasha Saad Aldoury, Nadia M.G. Al-Saidi, Rabha W. Ibrahim, Hasan Kahtan

**Affiliations:** a Department of Radiology Techniques, AL-Salam University College, Baghdad, 10064, Iraq; b Department of Applied Sciences, University of Technology, Baghdad, 10066, Iraq; c Near East University, Mathematics Research Center, Department of Mathematics, Near East Boulevard, PC:99138, Nicosia, Mersin 10, Turkey; d Department of Computer Science and Mathematics, Lebanese American University, Beirut, Lebanon; e Information and Communication Technology Research Group, Scientific Research Center, Al-Ayen University, Thi-Qar, Iraq; f Cardiff School of Technologies, Cardiff Metropolitan University, Western Avenue, Cardiff, CF52YB, The United Kingdom

**Keywords:** Fractional calculus, K-symbol, Mittag-Leffler functions, Caputo fractional differential operator, X-ray, Image enhancement, X-Ray Images Enhancement

## Abstract

Many image-processing applications heavily depend on the quality of medical images. Due to the unpredictable variation in the captured images, medical images frequently have problems with noise or low contrast; therefore, improving medical imaging is a challenging task. For better treatment, physicians need images with good contrast to provide the most detailed picture of the disease. The generalized k-differential equation based on the k-Caputo fractional differential operator (K-CFDO) is used in this study to determine the energy of the image pixels to improve the visual quality and provide a clearly defined problem. The logic behind using the K-CFDO approach in image enhancement is the ability of K-CFDO to efficiently capture high-frequency details using the probability of pixels as well as preserve the fine image details. Moreover, the visual quality of X-ray images is improved by performing a low-contrast X-ray image enhancement.

• Determine the energy of the image pixels for better pixel intensity enhancement.
• Capture high frequency image details using the image probability of pixels.

The findings of this study indicate that the average Brisque, Niqe, and Piqe values for the provided chest X-ray were found to be (Brisque=23.25, Niqe=2.8, Piqe21.58), and for the dental X-ray, they were (Brisque=21.12, Niqe=3.77, Piqe=23.49). The results of this study show potential improvements with the proposed enhancement methods that may contribute to increasing efficiency in healthcare processes at rural clinics. Generally, this model improves the details of medical images, which may aid medical staff throughout the diagnostic process by increasing the efficiency and accuracy of clinical decisions. Due to the improper setting of the suggested enhancing parameters, the current study included a limitation on image over-enhancement.

## Graphical abstract

**Figure:**
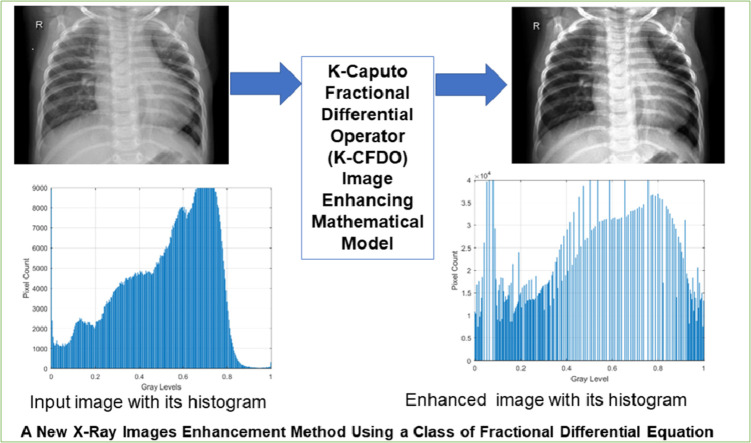

Specifications table

**Table:** 

Subject area:	Computer science
More specific subject area:	Image processing
Method name:	X-Ray Images Enhancement
Name and reference of original method:	N/A
Resource availability:	N/A

## Introduction

Medical image techniques have become increasingly used in today's healthcare systems for precisely identifying disorders and performing automatic diagnosis. One of the most common types of medical imaging is the X-ray image. Medical X-ray images are a significant and valuable source of studies and diagnostics for diseases because they are inexpensive and widely available. X-rays are a type of electromagnetic wave radiation that produces images of the inside of the human body in various hues of black and white. X-ray images have numerous applications in various real-life situations. The X-rays pass through the human body, penetrate the epidermis, and cross the bone segment, producing a brighter zone around the bone-like structure. The most prevalent applications include the detection of broken bones within the human body and aiding doctors in medical diagnostics.

Medical imaging recognizes various forms of X-Ray images, including 'Mammography,' 'Computed tomography (CT) scans,' and 'Fluoroscopy'. The mammography can help doctors detect breast cancer, but the CT scan uses a sequence of X-rays taken from different angles to provide doctors with more detailed images. The chest X-Ray images are used to diagnose such as COVID-19, lung cancer, and pneumonia [1]. Both X-Rays and CT scans are operated to visualize the disease moving across the lungs to understand the infection. Furthermore, X-Ray images can be used to assess pathologies such as kidney stones and orthopedic implants. X-Ray images are typically greyscale with a low level of intensity. The majority of acquired medical images have low contrast of tissues, making it difficult to discriminate between different tissues. As a result, medical image enhancement is a key stage in appropriately diagnosing a task.

Image enhancement is a method used in image processing to boost an image's quality. In order to enhance an image's visual appeal and make it better suited for analysis or display, it involves changing the pixel values in the image. Increasing the image's contrast, brightness, sharpness, and overall visual quality is the aim of image enhancement. A variety of techniques are used in image enhancement to boost an image's visual quality. Among the frequently employed methods are:

• Histogram equalization is a method for modifying an image's contrast by redistributing its pixel values. It is frequently applied to improve the look of grayscale images [2].
• Contrast stretching: Stretching the range of pixel values in an image is known as "contrast stretching," which increases the contrast. It is frequently applied to enhance the visual appeal of low-contrast images [3].
• Sharpening: By enhancing the high-frequency content of an image, the sharpening technique makes an image sharper. It is frequently applied to enhance the clarity and detail of images [4].
• Edge enhancement: This method improves an image's edges to make them more noticeable. It is frequently applied to enhance the clarity and detail of images.

The use of image enhancement is widespread, including in satellite imaging, surveillance systems, and medical imaging. Image enhancement is used in medical imaging to improve the contrast of X-ray or MRI images to aid in diagnosis.

Medical image enhancement is one of the image processing categories that has a significant impact on diagnosis judgments. Image contrast is an essential topic in medical imaging and refers to the lightest and darkest pixels in an image. The contrast between the image and the border representation, on the other hand, is comparatively inadequate and weak [5]. With the low-quality images, visually extracting features from these X-ray images is a difficult task. Some contrast enhancement models can be used to improve the quality of these images [3]. For image contrast enhancement, many image processing methods are being developed. The ability of fractional calculus has been extended in research and engineering [6,7].

Generally, capturing medical images might result in information loss and blurred or less useful images. The desire for improved medical imaging has risen to assist experts in making precise diagnoses. The responsibilities of enhancement in medical images are to increase the region of interest while keeping the features of an enhanced image [6], [7], [8], [9], [10]. Because a normal image is not clear and may not display useful information, or its brightness level appears inappropriate, we perform image enhancement to obtain a better and clearer image. As a result of the rapid growth in the usage and applications of medical images, it has become critical to create tools and methods for medical image processing. The object of this study is to provide a reliable and effective contrast enhancement technique for diverse X-ray images employing the k-symbol fractional calculus model to improve medical imaging so that doctors can make diagnoses more confidently and quickly [11], [12], [13], [14].

The objective of this paper is to propose the k-fractional differential equation and k-Mittag-Leffler functions as a useful tool to deal with X-ray images under diverse lighting situations, such as non-uniformly lighted and low-light X-ray images, by using an appropriate integral of arbitrary order ρ and the k-fractional differential. The novelty of this study is the new mathematical operation using the k-Caputo fractional differential operator (K-CFDO) that manipulates the pixel values in an image to improve its quality for accurate diagnoses.

## Proposed X-ray enhancement method

In this section, the pixel energy based on the K-Caputo fractional differential operator (K-CFDO) is defined first. Then a generalized energy K-fractional differential equation with K-CFDO is defined as well. Moreover, the upper bound solution of this equation is given by the K-Mittag-Leffler functions. Lastly, the coefficient values (connected values) of the upper bound solution are used to define the window to get the enhanced image.

### Methods

The field of image enhancement is one where fractional calculus has shown promise. Capturing long-range relationships, managing complicated textures, edge-preserving filtering, multiscale analysis, and adaptability to image properties are some advantages of employing fractional calculus in image improvement. Fractional calculus is useful for improving images because it can retain edges, manage complicated textures, record long-range relationships, and do multiscale analysis while also adapting to the properties of the image. Due to these benefits, fractional calculus is a useful tool for enhancing the visual appeal and readability of images in a variety of applications, including computer vision, satellite imaging, and medical imaging.

The methods described above investigated many approaches to image enhancement. These approaches work well for improving the overall image but not for improving sub-regions. Therefore, an effective image enhancement model for various X-ray images using the k-symbol fractional calculus is proposed in this study that can enhance and better display the image information than the traditional image enhancement models.

#### K-symbol concept

The k−symbol gamma function$\;\Gamma_{k}$, often known as the inspire gamma function, is expressed as tails [8]:

(1) $$\Gamma_{k}\left(\xi \right)=\underset{n\to \infty }{\text{lim}}\frac{n!k^{n}{\left(nk\right)}^{\frac{\xi }{k}-1}}{{\left(\xi \right)}_{n,k}},$$

Where ${\left(\xi \right)}_{n,k}≔\xi \left(\xi +k\right)\left(\xi +2k\right)...\left(\xi +(n-1)k\right)\;$and ${\left(\xi \right)}_{n,k}=\frac{\Gamma_{k}\left(\xi +nk\right)}{\Gamma_{k}\left(\xi \right)}.$

By using the above structure of k-symbol functions, it can be defined the K-Mittag-Leffler functions, as tails [9,10]

(2) $$\Xi_{k}^{\alpha ,\beta }\;\left(\xi \right)=\sum_{n=0}^{\infty }\;\frac{\xi^{n}}{n!\Gamma_{k}\left(n\alpha +\beta \right)\;}\;$$

#### K-Caputo fractional differential operator (K-CFDO)

Definition 1 Let m=1,2,.... Then for m−1<ρ<m and analytic function φ; the k-Caputo derivative of order ρ is expressed by [9]

(3) $${}_{k}^{c}D_{\xi }^{\rho }\varphi \left(\xi \right)=\frac{1}{k\Gamma_{k}\left(m-\;\frac{\rho }{k}\right)}\int_{0}^{\xi }\varphi^{\left(k\right)}\left(\zeta \right){\left(\xi -\zeta \right)}^{m-\;\frac{\rho \;}{k}\;-1}d\zeta ,$$

$$\rho \in \left(m-1,m\right);\;\;\frac{d^{m}}{d\xi^{m}}\varphi \left(\xi \right),\;\;if\;\;\;\rho =m.$$

The integral of arbitrary order ρ,whereR(ρ)>0 for a function φ(ξ), is

(4) $${}_{k}^{c}I_{\xi }^{\rho }\varphi \left(\xi \right)=\left(\frac{1}{k\Gamma_{k}\left(\frac{\rho }{k}\right)}\right)\left(\int_{0}^{\xi }\varphi \left(\zeta \right){\left(\xi -\zeta \right)}^{\frac{\rho }{k}\;-1}d\zeta \right)\;;\;\;\;\;R\left(\rho \right)>0.$$

#### The generalized K-differential equation

In this method, we deal with the k-fractional differential equation of the form

(5) $${}_{k}^{c}D_{\xi }^{\rho }\varphi \left(\xi \right)=\;E\left(\varphi \left(\xi \right),\xi \right),$$

where E:Δ×Δ→Δ is indicated the enhanced image with E(0,0)=0. Our aim is to construct a real solution to Eq. (5) in terms of the k-Mittag-Leffler functions.

#### Upper bound solution

In this sub-section, we use the hypergeometric function to approximate the solution of Eq. (5).

Proposition 1

Assume that φ(ξ) belongs to the convex functions class in open unit disk. If $|{\left(\zeta^{n}\right)}^{\left(k\right)}|\leq \frac{k}{(n-1)!}.\;$Then

(6) $$\left|{}_{k}^{c}D_{\xi }^{\rho }\varphi \left(\xi \right)\right|\leq \;r^{1-\frac{\rho \;}{k}}\;\Xi_{k}^{1,\;1-\frac{\rho }{k}}\left(r\right)$$

where Ξ indicates the K-Mittag-Leffler function. Eq. (6) satisfies Koebe function.

(7) $$K\left(\xi \right)=\frac{\xi }{\left(1-\xi \right)},\;\xi \in \Delta .$$

Proof. Let $\varphi \left(\xi \right)=\sum_{n=1}^{\infty }\varphi_{n}\;\xi^{n}$ be a convex function. Then the coefficients satisfy $|\varphi_{n}|\leq 1$ for all n. Furthermore, we obligate the following inequalities

$$\left|{}_{k}^{c}D_{\xi }^{\rho }\varphi \left(\xi \right)\right|=\left|{}_{k}^{c}D_{\xi }^{\rho }\left(\sum_{n=1}^{\infty }\varphi_{n}\;\xi^{n}\right)\right|=\left|\sum_{n=1}^{\infty }\varphi_{n}\;{}_{k}^{c}D_{\xi }^{\rho }\left(\xi^{n}\right)\right|$$

$$=\left|\sum_{n=1}^{\infty }\varphi_{n}\frac{1}{k\Gamma_{k}\left(m-\frac{\rho }{k}\right)}\int_{0}^{\xi }{\left(\zeta^{n}\right)}^{\left(k\right)}{\left(\xi -\zeta \right)}^{m-\frac{\rho }{k}-1}d\zeta \right|$$

$$\leq \frac{k}{k\Gamma_{k}\left(m-\frac{\rho }{k}\right)}\;\sum_{n=1}^{\infty }\left|\varphi_{n}\right|\int_{0}^{\xi }\left|{\left(\xi -\zeta \right)}^{m-\frac{\rho }{k}-1}\right|d\zeta$$

(8) $$\leq \frac{k}{\;k\Gamma_{k}\left(m-\frac{\rho }{k}\right)}\;\sum_{n=1}^{\infty }\;\frac{1}{n!}\int_{0}^{\xi }\left|{\left(\xi -\zeta \right)}^{m-\frac{\rho }{k}-1}\right|d\zeta \;$$

When *m* = *n*, we have

(9) $$\left|{}_{k}^{c}D_{\xi }^{\rho }\varphi \left(\xi \right)\right|\leq 1/r^{\frac{\rho }{k}}\sum_{n=1}^{\infty }\frac{1}{\Gamma_{k}\left(n-\frac{\rho }{k}\right)}\frac{r^{n}}{\left(n-1\right)!}=1/r^{\frac{\rho }{k}}\sum_{n=0}^{\infty }\frac{r^{n+1}}{n!\Gamma_{k}\left(1+n-\frac{\rho }{k}\right)}=r^{1-\frac{\rho }{k}}\sum_{n=0}^{\infty }\;\frac{r^{n}}{n!\;\Gamma_{k}\left(1+n-\frac{\rho }{k}\right)}\;=r^{1-\frac{\rho \;}{k}}{\;\Xi }_{k}^{1,\;1-\frac{\rho }{k}}\left(r\right)$$

The proposed image enhancement method is based on a new mathematical operation, the k-Caputo fractional differential operator (K-CFDO), that manipulates the pixel values in an image to improve its quality.

The logic behind using the pixel energy based on the k-Caputo fractional differential operator (K-CFDO) in image enhancement is the ability of K-CFDO to efficiently capture high-frequency details using the probability of pixels as well as preserve the fine image details. In our application study, the probability of the pixel is expressed by a value of 0 < *r* < 1.

The enhanced image In(i,j) is given by:

(10) $$In\left(i,j\right)=\sum_{i=1}^{n}\sum_{j=1}^{m}I\left(i,j\right)\;\times \frac{r_{\left(i,j\right)}^{1-\frac{\rho }{k}}}{\Gamma_{k}\left(2-\frac{\rho }{k}\right)}\;$$

Where I(i,j) is the input image, r is the pixel probability, and k chosen experimentally equal to 2.

### Algorithm I

The algorithm steps:

1. Consider the source image (*In*).
2. Set the parameters of ρ and k.
3. Determine the pixel's probability value (r).
4. Using Eq. (9) to determine the proposed K-CFDO
5. Calculate the enhanced image using Eq. (10).

**Fig. 1:**
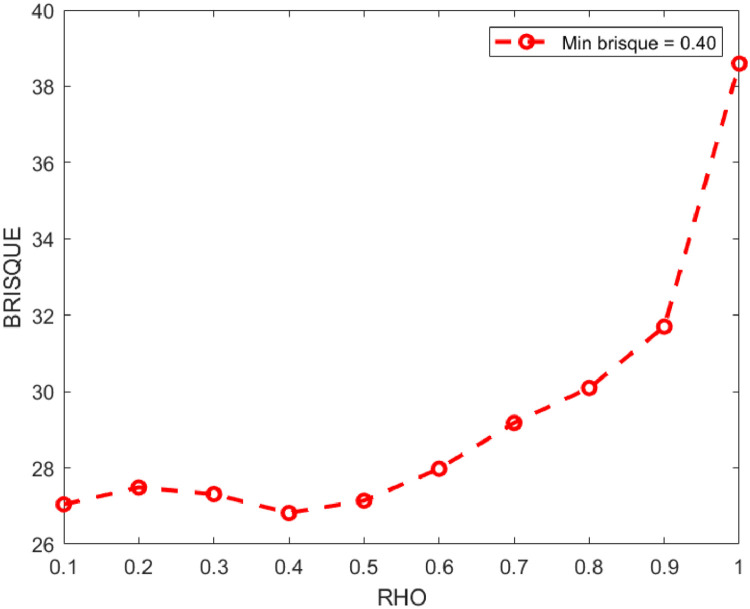
The BRISQUE score for fractional parameter (ρ) values.

The parameter ρ is used for fine image enhancement and it is chosen empirically, as shown in Fig. 1. It has been discovered that when the value of ρ is equal to 0.4, the best brisque score is produced (lower is better), due to fact that the proposed K-CFDO has efficiently improved image details while preserving fine features of the input image.

**Fig. 2:**
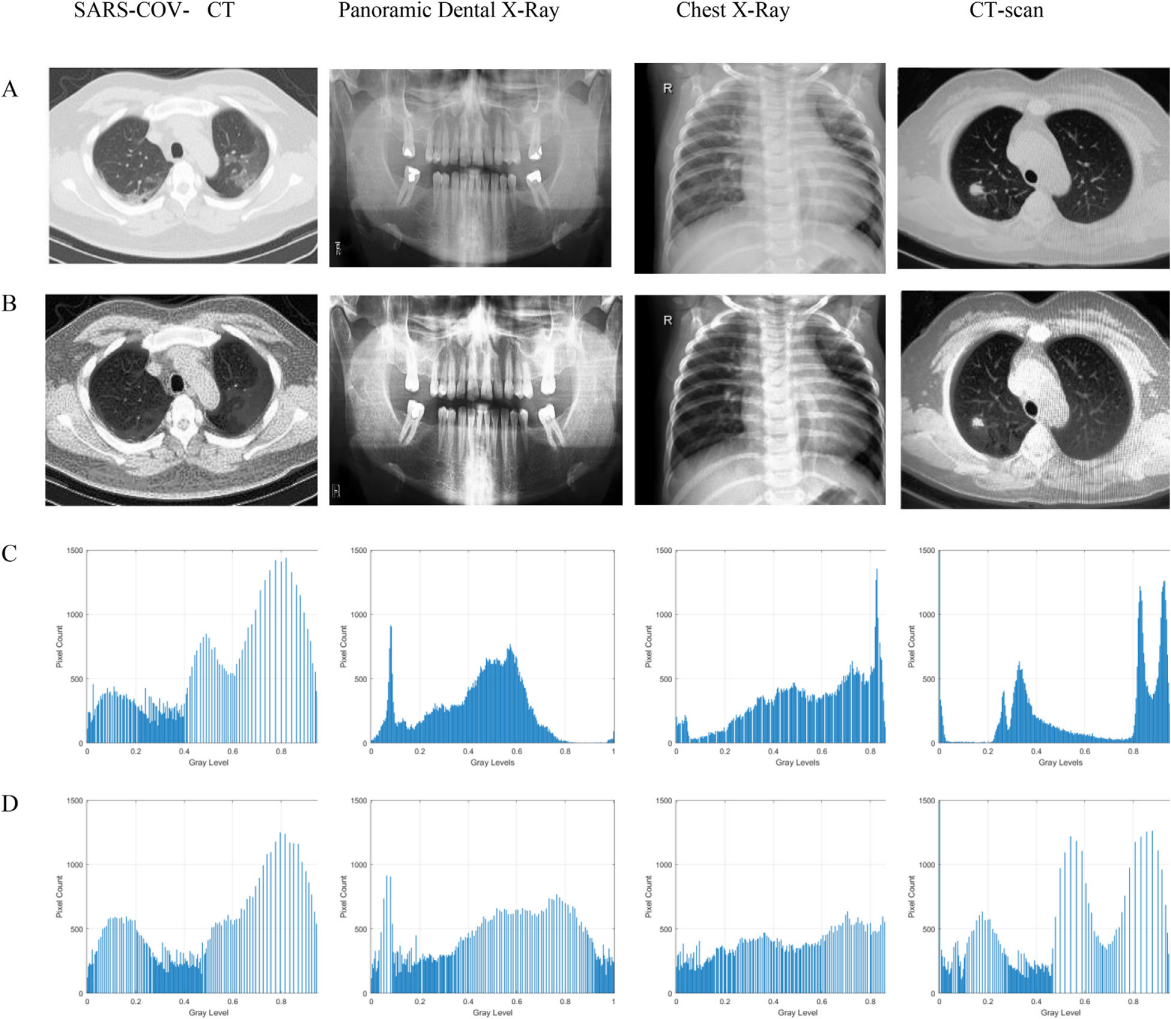
The input enhanced images and the histogram results. (A) Input image, (B) Enhanced image, (C) Input image histogram of, (D) The enhanced image histogram.

Fig. 2 depicts the qualitative outcome of the proposed enhancement model. The loss of image details in the input images is easily visible in Fig. 2(A). However, as compared to the suggested method, the contrast of the source images is stretched, as seen in Fig. 2. (c). Furthermore, areas lacking details grow brighter and more distinct after the improvement procedure. The histogram analysis shows the differences in the characteristics of the input and improved images. Fig. 2(C) shows the compactness of the pixel probability distribution in the input images, whereas the pixel intensities distribution in the enhanced image is more spread. This suggests that both the image quality and the contract have been improved in all enhanced images.

The benefit of using the k-symbol fractional calculus model for medical image enhancement is that it can improve low contrast intensities by calculating the coefficient values (connected values) of the upper bound solution that are convoluted with the input image.

## Results

The proposed algorithm's code and comparison with other approaches are written in MATLAB 2021b.

### Image dataset

In this study the following publicly available X-Ray medical image datasets are used:

1- SARS-COV-2 CT-Scan dataset [11]. SARS-CoV-2 CT images from a large number of real patients.
2- Panoramic dental X-Rays dataset [12]. The image dataset consists of 116 patients' anonymized and deidentified panoramic dental X-rays.
3- Chest X-Ray Images (Pneumonia)dataset [13]. The large image collection is made up of two types of Chest X-Ray images (Pneumonia).
4- The CT scan dataset [14]. The large COVID-19 CT Scans collection is made by the Italian Society of Interventional and Medical Radiology.

### Performance metrics

Three no-reference/blind image quality assessment measures are utilized to evaluate the proposed image enhancement model: ``the blind no-reference image spatial quality evaluator (BRISQUE) [15], ``the naturalness image quality evaluator (NIQE) no-reference image quality score'' [16], and the ``perception-based image quality evaluator (PIQE) . These image assessments measure the image quality without a reference image by predicting the visual image quality. The BRISQUE compares the input image to various natural scene images. The BRISQUE metric is based on a subjective quality score. The NIQE calculates the quality score for an image using arbitrary distortion. PIQE uses a block-wise approach to calculate the quality of a given image based on an arbitrary distortion. The lower scores indicate better image quality.

**Fig. 3:**
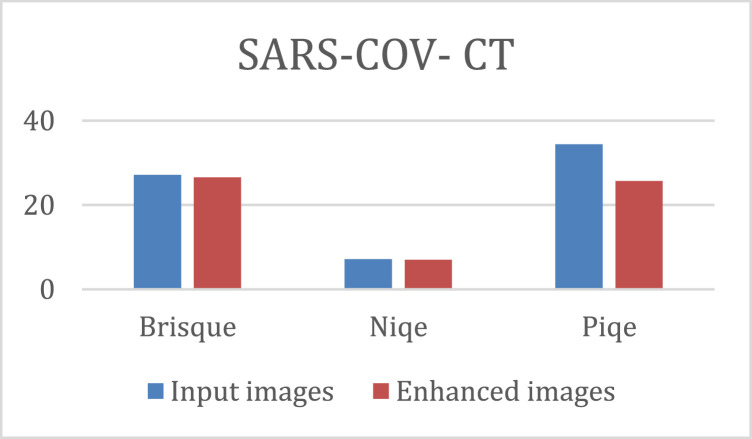
SARS-COV-2 CT-Scan Dataset [11].

**Fig. 4:**
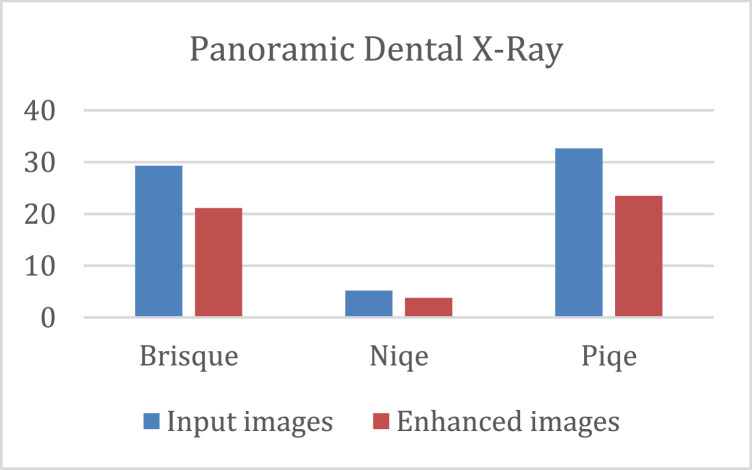
Panoramic Dental X-rays [12].

**Fig. 5:**
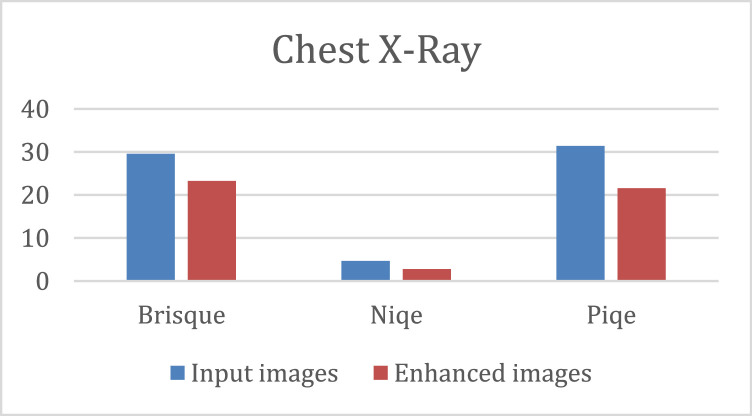
Chest X-Ray Images (Pneumonia) [13].

**Fig. 6:**
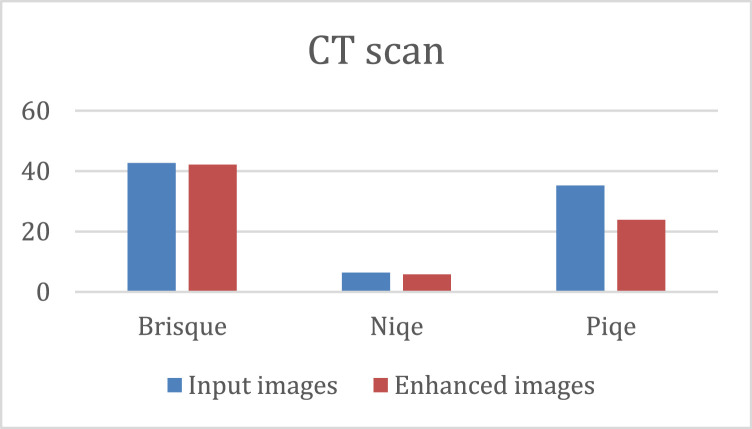
The CT scan dataset [14].

The quantitative results of the K-CFDO proposed image enhancement using Brisque, Niqe and Piqe are illustrated in Fig. 3, for SARS-COV-CT-Scan image dataset, Fig. 4, for panoramic dental X-Rays, Fig. 5, for Chest X-Ray images, and Fig. 6, for CT scans image dataset.

**Fig. 7:**
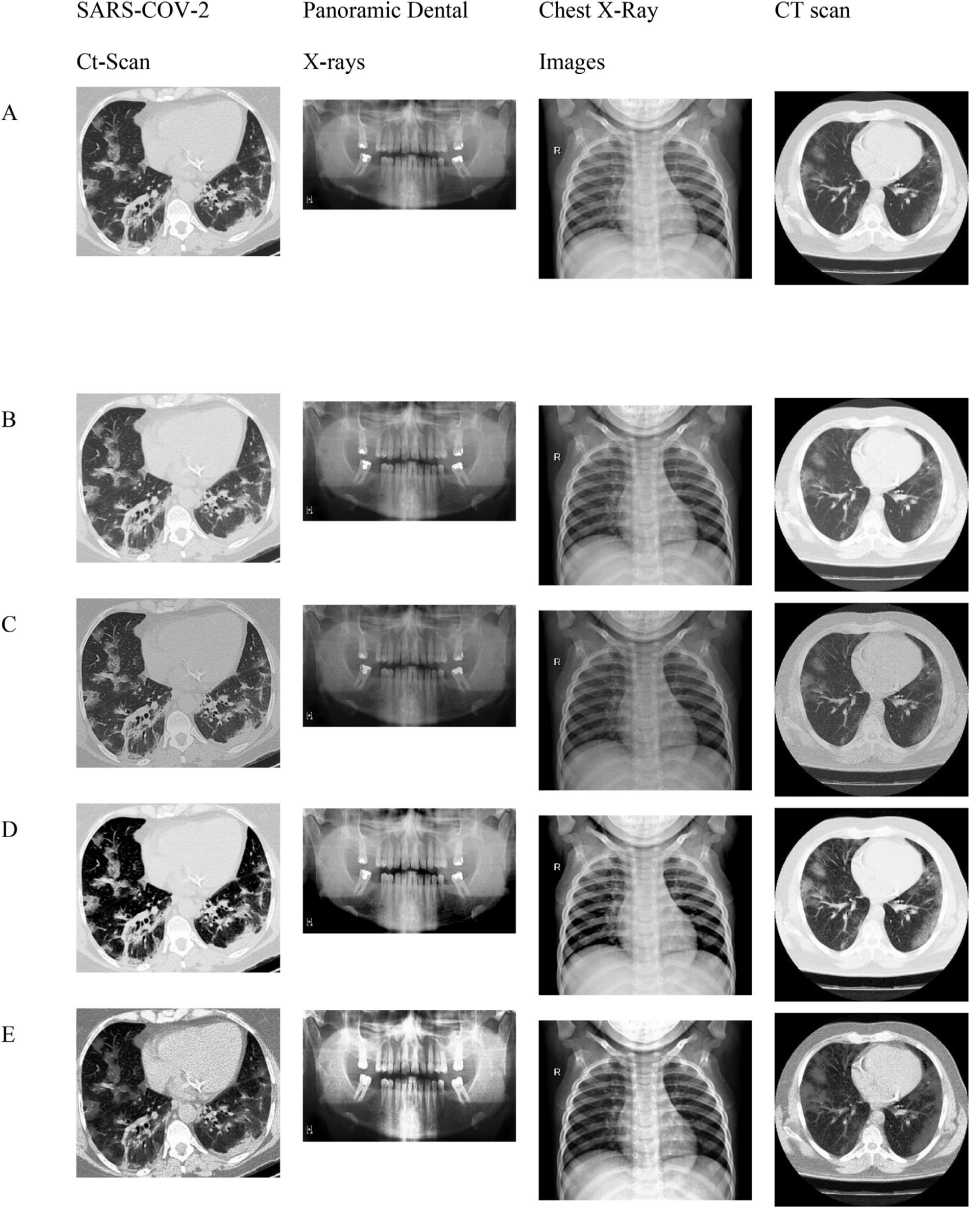
The proposed and other models results.(A) Input image, (B) Al-Shamasneh et al. [17], (C) Raghunandan et al. [18]., (D) Z. Al-Ameen [19], (E) Proposed method.

It should be noted that lower Brisque and Niqe scores indicate higher image quality. The overall results of the four image datasets (for SARS-COV-CT-Scan, panoramic dental X-Rays, Chest X-Ray, and the CT scans) for the datasets of the Brisque, Niqe and Piqe as most common image quality metrics reported in previous studies. The Brisque of SARS-COV-2 CT-Scan dataset was rated as the lowest among all Brisque metrics for all four image datasets, because of homogeneous intensity distribution despite the sharp edges and high intensity of lung region with respect to the surrounding tissues (Similarly, for the Niqe metric in Chest X-Ray Images (Pneumonia) dataset). For the comparative analysis, we used the following existing approaches to demonstrate that the suggested enhancement model is effective as a medical image improvement tool: Al-Shamasneh et al. [17] developed a fractional entropy-based improvement approach for kidney images. Raghunandan et al. [18] developed an image enhancement approach for license plates based on the Riesz fractional operator. Al-Ameen [19] developed a strategy for enhancing MRI brain imaging. All the above procedures were run on the same machine. Fig. 7 depicts the qualitative results of the proposed and other approaches for four image datasets. When the enhancing outcomes of existing methods are compared to the proposed method, the proposed method outperforms the existing methods in terms of image quality.

The proposed method captures high frequency image details despite blur and distortion caused by image capturing devices. By using the coefficient values (connected values) of the upper bound solution of K-Mittag-Leffler functions the proposed method boosts the dark portions of input images while keeping the bright areas. Generally, the proposed model's makes the image edges well-defined and due to its ability in capturing all high frequency features. The proposed method yields acceptable visual results for images with low illumination. Table 1 shows the quantitative assessments of the proposed and the existing models. Although the proposed method obtains the best Brisque and Niqe for practically all of the images in the four datasets, its performance drops marginally when compared to Al-Shamasneh [17]. This could be due to the image complexity compared to those identified in the other two datasets, and it could be a major drawback of the current study.

**Table 1:** Evaluation of several image enhancing techniques and the proposed model (BQ is Brisque score and, NQ is Niqe score.

	**SARS-COV- CT**		**Panoramic Dental X-Ray**		**Chest X-Ray**		**CT scan**	
	BQ	NQ	BQ	NQ	BQ	NQ	BQ	NQ
Input images	27.16	7.15	29.29	5.17	29.57	4.70	42.6	6.4
Al-Shamasneh [17]	27.30	7.08	29.56	5.27	29.67	4.80	42.6	6.5
Raghunandan [18]	43.08	11.06	42.80	8.68	38.81	5.92	42.4	8.8
Al-Ameen [19]	28.43	8.38	29.71	5.09	34.02	4.69	42.6	6.2
Zhang [21]	–	–	–	–	14.71	–	41.7	–
Fu [20]	27.68	8.95	28.36	4.21	23.53	3.85	44.2	3.6
Proposed	26.56	7.02	21.15	3.78	21.12	3.77	42.6	5.8

According to the subjective evaluation results, the proposed model shows an excessive enhancement in brightness of input images, as shown in Fig. 7. In addition, Table 1 shows that, when compared to other methods, the proposed method produces more detailed images. As shown in Table 1, the Brisque and Niqe values of the proposed model are the lowest, which clearly shows that proposed model can present higher contrast, and the best enhancement effect. Table 1 shows that the proposed enhancement method outperforms the existing models in terms of Brisque and Niqe scores. Zhang et al. [29] proposed dual illumination estimation for image exposure correction. This technique produced 14.71 Brisque for Chest X-Ray, which is better than the proposed method's Brisque. This accomplishment is because this approach was developed to estimate image exposure adjustment. However, this approach did not exhibit the ability to preserve image features for CT scans. For almost all the images in the four datasets, the proposed method gets the best Brisque and Niqe. However, for CT lungs images, the proposed method's Niqe score increased marginally when compared to Fu et al. [20]. The reason for this is this method was created to enhance general images. Regardless of dataset or content, the proposed technique gets the best Brisque and Niqe scores (lower is better). Because of its consistent results across different datasets, the proposed method outperforms the other methods. However, because they were built with a specific application, some existing procedures may give greater results when employed under specific conditions.

## Conclusion

Image enhancement is crucial to the medical community's efforts in correctly diagnosing diseasesThe method described in this paper can automatically improve the contrast of various X-ray images. In this study, a novel method for enhancing X-Ray medical images based on k-Caputo fractional differential operator (K-CFDO) is proposed. The proposed enhancement model successfully deals with images under diverse lighting situations, such as non-uniformly lighted and low light images, by using an appropriate integral of arbitrary order ρ and the k-fractional differential. The proposed method using an image processing approach based on new mathematical operation that manipulates the pixel values in an image to improve its quality for accurate diagnoses, better treatment planning, and enhanced patient care. The improved image achieves a decent trade-off between boosting brightness, increasing contrast, and retaining naturalness. The experimental results on four medical image datasets demonstrated that the suggested approach outperforms existing methods for image enhancement in general. The current study included the image over-enhancement limitation, caused by the unsuitable setting of ρ and the k parameters. Future work may adjust the current model for specific applications to maximize the advantage of enhancement.

## Ethics statements

The platforms’ data redistribution policies were complied with.

## CRediT authorship contribution statement

**Rasha Saad Aldoury**: Validity tests, Writing- Original draft preparation. **Nadia M. G. Al-Saidi**: Software, Validation. **Rabha W. Ibrahim:** Conceptualization, Methodology, Software. **Hasan Khtan**: Material preparation, data collection and analysis.

## Declaration of Competing Interest

The authors declare that they have no known competing financial interests or personal relationships that could have appeared to influence the work reported in this paper.

## Data Availability

No data was used for the research described in the article.
